# School Nurses on the Front Lines of Healthcare: The Approach to Maintaining Student Health and Wellness During COVID-19 School Closures

**DOI:** 10.1177/1942602X20935612

**Published:** 2020-06-25

**Authors:** Rachel Rothstein, Robert P. Olympia

**Affiliations:** Penn State University College of Medicine, Hershey, PA; Professor, Departments of Emergency Medicine and Pediatrics, Penn State College of Medicine, Attending Pediatric Emergency Medicine physician, Penn State Hershey Medical Center, Hershey, PA

**Keywords:** coronavirus, COVID-19, pandemic, social distancing, school closures

## Abstract

In response to the novel coronavirus disease 2019 (COVID-19) pandemic, most states in the United States enacted statewide school closures, ranging in duration from 1 month to the remainder of the academic year. The extended durations of these closures present unique challenges, as many families rely on the school as a source of physical activity, mental health services, psychosocial support, child care, and food security. While the school doors may be closed, the school nurse can still play a vital role in emergency management. This article discusses challenges and proposes solutions to maintaining student health and wellness during extended school closures due to the COVID-19 pandemic. Furthermore, it is inevitable that until a vaccine for coronavirus is developed and readily available, many schools will continue to see future closures, though likely for shorter periods of time, as they respond to local outbreaks.

## Who Is the ER Pediatrician?

Dr. Robert Olympia, MD, is a pediatric emergency medicine physician with more than 20 years of experience, currently working in an emergency department in the “sweetest place” on Earth (Hershey, PA). He is a professor in the Departments of Emergency Medicine and Pediatrics at the Penn State University College of Medicine. His research interests include emergency and disaster preparedness for children in the setting of schools and school-based athletics, as well as in sports-related illnesses and injuries. He has presented his research both regionally and nationally and has lectured on a variety of topics pertaining to pediatric emergency medicine, such as fever and infectious diseases, trauma, sports-related injuries, and disaster preparedness.

## Who Is Dr. Olympia’s Coauthor?

Rachel Rothstein is a fourth-year medical student at the Penn State University College of Medicine and a recent graduate of Tufts University School of Medicine Master of Public Health Program. Her interests include pediatrics, pediatric surgery, and public health.

## What Is the Purpose of the “School Nurses on the Front Lines of Healthcare” Series?

The “School Nurses on the Front Lines of Healthcare” series will present cases reflecting emergencies commonly encountered in the school setting, focusing on an evidence-based approach to the initial management, stabilization, and disposition of the ill or injured child.

Special features unique to each article are Extra Credit Points and Report Cards. Extra Credit Points are trivia questions or clinical pearls scattered throughout the article related to the topic at hand. Report Cards are concise tables summarizing key points in each article that you can photocopy and laminate or photograph and keep on your smart device for easy access.

## Case

During an otherwise normal afternoon in early March, your superintendent urgently assembles the school nurses, principals, and other key faculty members, as well as local public health officials, to discuss the growing threat of COVID-19. Your team ultimately decides to close the schools for the next 2 weeks, effective immediately. Shortly thereafter, your state governor declares a state of emergency and announces statewide closure of schools for a minimum of 6 weeks.

Parents in the community begin contacting your team with concerns. Many parents are essential workers and cannot obtain child care. Others are concerned about food security, in the absence of school meal programs. The list of student health and wellness concerns continues. Your school district is fortunate to have an emergency procedure in place for infectious disease outbreaks, and the school closure provides you and the school staff time to review and update your emergency and disaster plan, addressing many aspects of wellness for your students and their families.

## School Closures During Historical Infectious Disease Outbreaks

While the term *social distancing* is likely new to many, the concept of school closures as a tool for reducing spread of infectious diseases originated long before the COVID-19 pandemic. During the 2009-2010 influenza H1N1 pandemic, over 720 schools, housing nearly 368,300 students, closed across the United States (Klaiman et al., 2011). While the current durations of school closures exceed those of other infectious disease outbreaks in recent history, consistent are the challenges to maintaining student health and wellness in light of closures.

## The Role of the School Nurse During School Closures

The National Association of School Nurses (NASN) recognizes the school nurse as a “vital member of the school team, who collaborates with community agencies to develop comprehensive emergency response procedures” (NASN, 2019, para. 1). Likewise, the school nurse provides expertise in school health as a “leader and integral partner with school staff and outside agencies in developing comprehensive school plans/procedures” (NASN, 2019, para. 3). NASN is continually providing up-to-date resources on COVID-19 for school nurses, which incorporate emerging information from the Centers for Disease Control and Prevention (CDC) and other groups (CDC, 2020b; NASN, 2020). Furthermore, the uncertainty surrounding future “peaks” of COVID-19 or other infectious disease outbreaks emphasizes the importance of emergency and disaster planning.

As school districts enter this unfamiliar phase of emergency management, the school nurse plays a critical role in addressing student health and wellness during school closures. The document, “Considerations for School Nurses When Providing Virtual Care,” highlights the role of virtual care in addressing student needs, continuing case management, providing resources to families, and mitigating health disparities (NASN, 2020). NASN also provides, “Guidance for School Nurses to Safely Send and Receive Resources Between School and Home During COVID-19,” to ensure safe transfer of both student and school property, including backpacks, student medication, school supplies, electronic devices, and ongoing food services to socially and medically disadvantaged families (NASN, 2020). Finally, “Ideas for School Nurse Activities during the COVID-19 Pandemic” provides a list of activities that school nurses can complete in light of closures (NASN, 2020).

Regarding school reopenings, NASN provides template letters, addressing state leaders, local leaders, and school superintendents, advocating for the inclusion of school nurses in planning processes (NASN, 2020). In the article, “Interim Guidance: Role of the School Nurse in Return to School Planning,” NASN outlines school nurse roles according to the five major principles of the *Framework for 21st Century School Nursing Practice™* (Care Coordination, Community/Public Health, Leadership, Quality Improvement, and Standards of Practice). For school nurses practicing in schools that are open, NASN offers “Considerations for School Nurses Regarding Care of Student and Staff that Become Ill at School or Arrive Sick” and “Facemask Considerations for Healthcare Professionals in Schools” (NASN, 2020).

## Challenges to Obtaining Child Care Coverage

Currently, 16 sectors of the workforce qualify as “critical infrastructures” and are essential to the country’s continued functioning, potentially leaving 45.9 million children, who otherwise would be in school, without adequate supervision (Bayham et al., 2020). While there is no definitive age at which it is appropriate to leave a child home alone, many resources are available to guide parents in this decision. Leaving children home alone may present risks to both physical health and mental well-being.

On the national level, the Families First Coronavirus Response Act includes a provision that expands protection for employees that during a public health emergency are unable to work due to a need for leave to care for their child because the school or day care has been closed or the child care provider is unavailable (Moss et al., 2020). Similarly, the Coronavirus Aid, Relief and Economic Security Act includes a $3.5 billion expansion in funding of the Child Care and Development Block Grant, to help states establish child care assistance for essential workers (Johnson-Staub, 2020). Examples of such interventions include temporary pandemic child care centers in Ohio and regional enrichment centers in New York City. To assist your community’s essential workers in obtaining child care coverage, view your state’s emergency child care provisions and actions in response to COVID-19 on the Hunt Institute website in Table 1. Also, the Khan Academy websites listed in Table 1 offer free online video classes, including music, games, crafts, and educational materials, for children to follow along while parents work or accomplish tasks in the home.

**Table 1.** Report Card: Resources to Aid Families in Your Community in Maintaining Health and Wellness

Child care	Hunt Institute	Information on your state’s emergency child care provisions to help essential workers obtain child care coverage	http://www.hunt-institute.org/covid-19-resources/state-child-care-actions-covid-19/
	Khan Academy	Virtual classes (e.g., music, games, crafts, educational) to engage children while parents complete work or tasks in the home	https://www.youtube.com/channel/UC2ri4rEb8abnNwXvTjg5ARw https://wideopenschool.org/ https://learn.khanacademy.org/khan-academy-kids/
Physical activity	Healthy Children	List of individual and family-oriented outdoor physical activities that follow social distancing guidelines	https://www.healthychildren.org/English/health-issues/conditions/chest-lungs/Pages/Getting-Children-Outside.aspx
	Online PE Network	Grade-specific physical activities for the home; weekly and monthly schedules to make exercise a part of children’s routines	https://openphysed.org/activeschools/activehome
	GoNoodle	Online physical activity and meditation videos for children to follow	https://family.gonoodle.com/
Food security and healthy eating	No Kid Hungry, USDA	Resources on the state and district levels to assist families in maintaining food security during the pandemic	https://www.nokidhungry.org/blog/heres-how-youre-supporting-hungry-kids-affected-coronavirus-state https://www.fns.usda.gov/meals4kids
	Feeding America	List of local food banks in your community, as well as information on their responses to the pandemic	https://www.feedingamerica.org/find-your-local-foodbank
	Nutrition	Strategies to make nutrition a priority during the pandemic	https://nutrition.org/making-health-and-nutrition-a-priority-during-the-coronavirus-covid-19-pandemic/
	CDC	General tips on promoting healthy eating in the home	https://www.cdc.gov/nccdphp/dnpao/features/national-nutrition-month/index.html
	Eat Right	Healthy recipes that children enjoy Safe opportunities to engage children in the kitchen	https://www.eatright.org/kids-eat-right-listing https://www.eatright.org/food/planning-and-prep/cooking-tips-and-trends/toddler-and-preschooler-tasks-in-the-kitchen
Mental health and well-being	Child Mind Institute	Strategies to improve sleep hygiene	https://childmind.org/article/encouraging-good-sleep-habits/
	National Hotline	Advocates, available to provide support and access to community resources for victims of domestic violence	https://www.thehotline.org/ Call 1-800-799-7233 or Text “LOVEIS” to 22522
	NASN	Guidance for parents on how to help children cope with COVID-19	https://www.nasn.org/nasn/nasn-resources/practice-topics/covid19
	Child Mind Institute	Guidance for parents on how to discuss COVID-19 with children	https://childmind.org/article/talking-to-kids-about-the-coronavirus/
	CDC	Activity and coloring workbooks to help facilitate COVID-19 discussions between parents and young children	https://www.cdc.gov/cpr/readywrigley/documents/RW_Coping_After_a_Disaster_508.pdf https://www.cdc.gov/childrenindisasters/pdf/coping_activity_page_english-p.pdf
	Crisis Text Line	Crisis counsellors, available to discuss COVID-19 and other related concerns with children	https://www.crisistextline.org/ Text “HOME” to 741741
Daily routine	Khan Academy	Comprehensive daily schedules and academic materials by grade level	https://keeplearning.khanacademy.org/daily-schedule#Preschool1

*Note*. PE = physical education; USDA = U.S. Department of Agriculture; CDC = Centers for Disease Control and Prevention.

## Extra Credit Point: How Many Hours of Physical Activity Should Children Get Each Day?

The CDC recommend that all children aged 5 to 17 years participate in 1 hour of moderate to vigorous physical activity each day, with an emphasis on aerobic activities such as walking, running, or biking, and that all children 3 to 5 years old participate in 3 hours of physical activity daily, through activities spread over the course of the day (CDC, 2020c).

## Challenges to Maintaining Physical Activity

In addition to maintaining a healthy body weight and fostering musculoskeletal, cardiovascular, and mental health, physical activity in children is crucial to “self-expression, building self-confidence, social interaction and integration” (World Health Organization, 2015, para. 13). Schools help children incorporate physical activity into their daily routines through participation in physical education (PE), recess, sports, and other extracurricular activities.

As a result of school closures, children lose access to these structured and supervised sources of physical activity. The extended duration of school closures resembles summer recess, a period during which physical activity levels decline and sedentary time and body mass index rise (Brazendale et al., 2017); Unlike summer recess, the pandemic limits children’s access to alternative sources of physical activity in the community, due to closures of public spaces such as parks, sports fields, tracks, and gyms. Many families lack resources and equipment in the home for children to continue their usual activities. Finally, social distancing limits the social interaction and bonding that physical activity often affords children.

Prior to the COVID-19 pandemic, nearly 32% of children in the United States were overweight or obese, and children spent an average of 7.5 hours in front of a screen per day (Brazendale et al., 2017; CDC, 2018). Both statistics are likely to rise in light of the pandemic. Families now have a critical opportunity to adopt new attitudes and strategies to make physical activity a priority. To provide families in your community with outdoor physical activities that follow social distancing guidelines, visit the Healthy Children website in Table 1. The Online PE Network website in Table 1 provides a list of grade-specific physical activities for the home, as well as monthly and weekly calendars to make these activities a part of children’s daily routines. Finally, in place of traditional screen time, the GoNoodle website in Table 1 provides free online physical activity and mindfulness videos for young children to follow.

## Challenges to Maintaining Food Security and Healthy Eating

Approximately 29.7 million children in the United States participate in the National School Lunch Program, which provides discounted or free school lunches to children of low-income households (U.S. Department of Agriculture [USDA], 2019). Similarly, the School Breakfast Program provides school breakfast to nearly 14.6 million children in the United States. (USDA, 2019). Food insecurity disproportionately affects children residing in rural and large urban areas, as well as single-parent, Black and Hispanic households (No Kid Hungry, 2020). Childhood hunger can contribute to malnutrition, difficulty concentrating, impaired academic performance, stress, poor social skills, and other adverse physical and mental health outcomes (No Kid Hungry, 2020). As a result of school closures, children lose access to these routine daily sources of meals. As of May 1, 2020, children in these programs have missed a collective one billion school meals (No Kid Hungry, 2020). To locate resources to help families in your community maintain food security, visit the No Kid Hungry and USDA websites in Table 1.

Even children residing in food secure households may encounter difficulty in maintaining healthy eating during school closures. For many children, school lunches provide the most nutritious meals of the day, by ensuring inclusion of fruits, vegetables, and milk. During school closures for summer recess, children typically consume fewer fruits, vegetables, and milk and more added sugars, saturated fats, and sodium (Brazendale et al., 2017). Unlike summer recess, the pandemic presents unique barriers to obtaining healthy foods, due to grocery store closures, delivery delays, and item shortages, while access to “fast foods,” which typically contain more saturated fat, sugar, salt, and calories, remain readily available. In efforts to expand reach during the pandemic, food banks are creating drive-through food distribution centers, preparing and distributing meals to children, prematurely opening summer meal programs, and keeping existing after-school meal programs accessible. To locate a food bank in your community and to learn more about your local food bank’s response to COVID-19, visit the Feeding America website in Table 1.

To provide your community with strategies to make nutrition a priority during the pandemic, visit the Nutrition website in Table 1. To provide families with healthy eating tips, visit the CDC’s website in Table 1. For a list of healthy recipes that children enjoy, visit the Academy of Nutrition and Dietetics’ Eat Right website in Table 1. The Eat Right campaign also helps parents find safe opportunities to engage their children in meal planning and preparation, which may help children become more curious about trying new foods.

## Challenges to Addressing Mental Health and Psychosocial Implications of Pandemic

Schools represent the largest providers of mental health services for children. Of the 16% of children in the United States receiving mental health services, over 70% do so in school, specifically through school counselors, nurses, psychologists, and social workers (Centers for Health and Health Care in Schools, 2012). In addition to counselling, schools provide crucial behavior management and crisis intervention services. As a result of school closures, children lose access to these critical resources. This is particularly concerning for children of racial and ethnic minority groups, who historically face greater difficulty in accessing mental health services. School closures also disrupt the structured daily routines that are critical for all children, especially those with underlying mental health and behavioral disorders. Finally, in social distancing from peers, children lose access to a critical social support network. Social isolation can further negatively affect mental health outcomes. School nurses searching for new strategies to address student mental health during the pandemic may schedule a zoom meeting with the Crisis Management Institute (2020).

The pandemic also presents unique psychosocial stressors, which may negatively affect the well-being of children and exacerbate underlying mental health disorders. Children may experience additional home stressors, as parents face unemployment, economic hardship, and difficulty balancing work with their caregiver responsibilities. Essential workers may experience greater stress in the workplace and spend less time with their children, which can negatively affect both the child’s well-being and the parent–child relationship. Physical activity, healthy eating, mindfulness, and sleep hygiene may all reduce psychosocial distress and improve well-being in children. To assist families in improving sleep hygiene, visit the Child Mind Institute website in Table 1. In addition to physical activity videos, the GoNoodle website in Table 1 also provides online meditation and mindfulness videos for young children to follow.

Over seven million cases of child abuse and neglect are reported in the United States each year, four children of which die each day (Safe Horizon, 2020). Professionals expect these numbers to increase in light of the pandemic, as household stress is a significant predictor of child abuse and neglect. In addition, victims have limited ability to seek refuge, due to closure of shelters, religious venues, and other community resources, as well as fear of contracting COVID-19 in the emergency department. Victims may connect with an advocate by visiting the National Hotline website in Table 1, by calling 1-800-799-7233 or by texting LOVEIS to 22522.

Children may experience the illness, hospitalization, and death of a loved one from COVID-19. These hardships may be difficult to understand based on developmental age and can have long-term psychiatric consequences. To provide families in your community with guidance on how to help children cope with COVID-19, visit the NASN website in Table 1. To engage young children in these discussions, age-appropriate COVID-19 activity workbooks and coloring books are available on the CDC websites in Table 1. Children may also discuss COVID-19 concerns with a crisis counsellor by visiting the Crisis Text Line website in Table 1 or by texting HOME to 741741.

## Creating a Daily Routine

School closures significantly disrupt children’s daily routines. Literature on the structured day hypothesis demonstrates that healthy behaviors are “beneficially regulated when children are exposed to a structured day (i.e., school weekday)” versus weekends and summer recess, through, “compulsory physical activity opportunities, restricting caloric intake, reducing screen time occasions, and regulating sleep schedules” (Brazendale et al., 2017, p. 1). Several nationally recognized organizations emphasize the importance of adhering to a structured schedule despite school closures. As an example, Table 2 displays a potential schedule for a late elementary school student, which incorporates resources mentioned in Table 1. The Khan Academy website listed in Table 1 provides more comprehensive daily schedules for students in preschool to second grade, third to fifth grades, sixth to ninth grades, and 10th to 12th grades, as well as academic lessons/assignments by subject for students who are not receiving ongoing remote education during school closures (Khan Academy, 2020). Finally, Gasol Foundation suggests creating themes for each weekday, to reinforce healthy behaviors and foster family time. Examples on this website include “Workout Wednesdays,” when family members participate in a family workout together, or “Feelings Friday,” when family members gather to discuss their emotions surrounding COVID-19 (Gasol Foundation, 2020). Families can utilize these resources to create a schedule that optimizes health and wellness during the pandemic.

**Table 2.** Report Card: Sample Daily Routine for Student Grades 3 to 5

Grades 3 to 5	
Morning	
8:00 to 9:00	Wake up, get dressed, wash up, eat breakfast
9:00 to 9:30	Start assigned schoolwork
9:30 to 10:00	Play outside or complete an Online PE Network physical activity (Table 1)
10:00 to 10:30	Continue assigned schoolwork
10:30 to 11:00	Reading time
11:00 to 11:30	Play outside or complete an Online PE Network physical activity (Table 1)
11:30 to noon	Continue assigned schoolwork
Afternoon/evening	
Noon to 1:00	Eat lunch
1:00 to 2:00	Continue assigned schoolwork
2:00 to 8:00	Relax, play outside, family time, help cook, eat dinner
8:00 to 9:00	Bedtime

*Note*. Adapted from Khan Academy (2020).

## For Schools That Have Reopened or Are Deciding to Reopen

The return to school introduces new challenges to maintaining student health and wellness. Several resources are available on the CDC’s, “Child Care, Schools and Youth Programs—Plan, Prepare, and Respond,” to assist schools, youth programs, camps sports and child care programs with the process of safely reopening or with maintaining safety for those that have already reopened (CDC, 2020a).

## Case Resolution

You visit the Hunt Institute website (Table 1) to learn more about your state’s emergency child care provisions in response to COVID-19. Likewise, you visit the No Kid Hungry and Feeding America websites (Table 1) to locate resources to assist your community in maintaining food security. You disseminate Tables 1 and 2, as well as these state- and community-specific resources, to parents in your community. You receive feedback from several families that these tools assisted them in adopting a new and healthy routine, despite school closures. Families in your community feel better equipped should the governor extend the duration of school closures. Moreover, you feel better prepared, should your school district encounter intermittent “peaks” of COVID-19 until a vaccine is developed and readily available, or an entirely new infectious disease outbreak occurs in the near future.

## Contact Dr. Olympia

If you have a clinical question, send your question to Dr. Olympia (rolympia@hmc.psu.edu). Questions will be selected and discussed as part of the “School Nurses on the Front Lines of Medicine” series. ■
